# Palliative Care as a Predictor of Professional Stress and Mental Health in Nurses: A Cross‐Sectional Study

**DOI:** 10.1002/nop2.70219

**Published:** 2025-09-11

**Authors:** Marija Ljubičić, Gianna Pavletić, Sonja Šare, Ivana Gusar, Boris Dželalija, Marijana Matek Sarić, Tatjana Šimurina, Samir Čanović, Dario Nakić, Suzana Konjevoda

**Affiliations:** ^1^ Department of Health Studies University of Zadar Zadar Croatia; ^2^ Department of Pulmonology General Hospital Zadar Zadar Croatia; ^3^ Medical School Ante Kuzmanića Zadar Zadar Croatia; ^4^ Department of Anesthesiology and Intensive Medicine General Hospital Zadar Zadar Croatia; ^5^ Department of Ophthalmology General Hospital Zadar Zadar Croatia; ^6^ Department of Nephrology General Hospital Zadar Zadar Croatia

**Keywords:** mental health, nurses, occupational stress, palliative care

## Abstract

**Aim:**

This study aimed to investigate the relationships among palliative care, occupational nursing stress and psychological indices.

**Design:**

This cross‐sectional study included 180 nurses employed in healthcare facilities (primary health care, outpatient clinics, hospitals, geriatric institutions and home care facilities) in the Zadar County, Croatia.

**Methods:**

We included two groups of nurses: those providing palliative care (*N* = 94) and those not providing palliative care (*N* = 86). Data on sociodemographic characteristics, personality types, self‐esteem, anxiety, depression, sleepiness, resilience and occupational nursing stress were also collected. Multiple linear regression models were used to determine the associations among palliative care, occupational nursing stress, resilience, sleepiness, anxiety, depression, self‐esteem and personality traits.

**Results:**

Palliative care was associated with nursing stress (*β* = 0.36; *p* < 0.001), death and dying (*β* = 0.28; *p* < 0.001), inadequate preparation (*β* = 0.14; *p* = 0.022) and sleepiness (*β* = −0.19; *p* = 0.021). Nurses providing palliative care reported a lack of support (*β* = −0.14; *p* = 0.028), which was associated with workload (*β* = 0.44) and uncertainty about patient care (*β* = 0.41), *p* < 0.001 for both. Conflict between nurses and physicians increased the conflict among nurses (*β* = 0.49; *p* < 0.001) and uncertainty about treatment (*β* = 0.35; *p* < 0.001). Death and dying contributed to workload (*β* = 0.23; *p* = 0.006) and uncertainty about treatment (*β* = 0.23; *p* = 0.014). Nursing stress contributed to depression (*β* = 0.16; *p* = 0.009) and anxiety (*β* = 0.12; *p* = 0.043) and vice versa. These results support the hypothesis that palliative nursing care is related to occupational stress.

**Conclusion:**

Providing palliative nursing care was associated with higher levels of occupational stress. Stress may trigger adverse health consequences such as the development of anxiety, depression and other chronic non‐communicable diseases. Support programs are indispensable for strengthening nurses' skills and reducing occupational stress to maintain their health and ensure high‐quality patient care.

**Implications for the Profession and/or Patient Care:**

A non‐probabilistic convenience sample of nurses was the subject of a cross‐sectional study to assess the level of occupational stress of nurses who provide palliative nursing care and its impact on nurses' mental health. Occupational stress in palliative care can have negative health consequences for nurses, including the development of anxiety, depression and other chronic non‐communicable diseases. Consequently, the quality of nursing care may be adversely affected. Palliative patients, especially at the end of life, are at risk of lower quality of healthcare, which can deteriorate care outcomes and patient satisfaction.

**No Patient or Public Contribution:**

This study included occupational nurses, not patients, service users, care‐givers or members of the public.

## 1 Introduction

An estimated 50 million adults worldwide and 4.4 million people in the European Region require palliative care, and this number is expected to increase in the near future (World Health Organization 2020, 2023). Palliative care is needed for a wide range of patients suffering from chronic diseases and other conditions with life‐threatening illnesses and uncertain or potentially fatal outcomes (World Health Organization 2020). The World Health Organization characterises palliative care as specialised medical care that aims to optimise quality of life and relieve patients' pain and suffering (World Health Organization 2020). A holistic interdisciplinary approach, patient‐centeredness, coordination and continuity of care are the most important goals that are essential for the best palliative care (Vočanec et al. 2022).

Palliative care involves a multidisciplinary approach involving physicians, nurses, psychologists, dieticians, physical therapists, occupational therapists, social workers, clergy, etc. (World Health Organization 2020). Nevertheless, nurses spend most of their time with patients and their families, especially in the case of a serious, incurable illness (Schroeder and Lorenz 2018). Palliative nursing care (PNC) includes support for individual physical, psychological, social and spiritual needs (Schroeder and Lorenz 2018). PNC reduces the stress, symptoms and anxiety of patients and their families during incurable illness, especially when the end of life is very near (Schroeder and Lorenz 2018). The emotional and stressful nature of PNC makes it difficult, as circumstances are heavily charged with emotion and the end of life is near. In addition, the nursing profession requires a high level of technical and scientific skills, the ability to work as part of a team, and to provide care 24 h a day, resulting in a variety of workplace stressors (Yu et al. 2023; Zhang et al. 2023). For these reasons, nurses may have stronger emotional experiences that may lead to higher perceptions of occupational stress (Cross 2019).

## 2 Background

Occupational stress is the subject of numerous scientific studies. A large body of evidence shows a relationship between occupational stress, job satisfaction and chronic diseases such as cardiovascular disease, hypertension, cancer, mental disorders and other diseases (Chang et al. 2021). As a complex negative condition, it occurs when the demands of the work environment are too high and the person is unable to cope with the excessive challenges (Abdoh et al. 2021). Indeed, professionals in certain fields of biomedicine are exposed to greater stressors at work. Nurses, for example, are in a constant state of tension between complex situations where they perform PNC and are at risk of developing occupational stress (Cross 2019; Kim and Kim 2020). These situations include the constant confrontation with the patient's suffering, the performance of painful procedures on the patient, the difficulty in relieving the patient's pain and unpleasant symptoms, and the feeling of helplessness when the patient's condition does not improve (Cross 2019; Kim and Kim 2020; May et al. 2022). Continuous emotional support, empathy imbalance and compassion fatigue, making professional and ethical decisions and dealing with the dying process and the death of the patient are additional stress factors in PNC (Cross 2019; Kim and Kim 2020; May et al. 2022). Dealing with the loss of a patient is confirmed to be one of the greatest challenges in clinical practice. In addition, insufficient sleep because of night shifts leads to nervousness, irritability and sleepiness the next day, which can affect the higher perception of stress and lower work efficiency of nursing staff (Ganesan et al. 2019). This can lead to the development of anxiety, demoralisation, poorer overall health and well‐being of the individual and low job satisfaction. Studies confirm that nurses have a high risk of developing mental illness and are prone to burnout symptoms (May et al. 2022). Some personal factors, such as personality and resilience, can contribute to these conditions (Yu et al. 2023; Zhang et al. 2023). Studies confirm that the reason why nurses in similarly stressful work situations suffer from burnout to varying degrees is related to personality factors (Angelini 2023). However, although the daily care of seriously ill patients, working in an environment where death and dying are present, as well as legal and bioethical dilemmas, play a role in burnout and loss of work motivation, the relationship between personality, resilience, stress and palliative care is not entirely known (Zanatta et al. 2020). In addition, resilience plays an important role in perceived social support and dimensions of burnout, so analysing its associations with palliative care and psychological stress can help develop ways to successfully adapt to challenging situations in palliative care (Zanatta et al. 2020; Zhang et al. 2023).

Although some studies confirm stress in palliative care, the assessment of occupational stress in nurses is complex (Cross 2019). Its associations with sleepiness, resilience, self‐esteem, personality, anxiety and depression are not entirely clear. It is not entirely clear whether the stress is related to the primary nursing job or the provision of PNC, which is confronted with death and dying more than other nursing fields, or whether it is due to personal factors. In addition, there is a dearth of studies comparing the perceived occupational stress of nurses who provide palliative nursing care with that of nurses who do not. These suggest the interesting hypothesis that nurses who provide palliative nursing care are at risk for adverse effects of chronic psychological stress. This study adds to the existing literature and focuses on the associations between perceived occupational stress, general stress, personality, anxiety and depression and offers opportunities for developing strategies to support nurses who provide PNC.

## 3 The Study

### 3.1 Aim of the Study

This study aimed to assess the level of occupational stress experienced by nurses who provide PNC compared with nurses who do not. In addition, it aimed to determine the associations between perceived occupational stress, general stress, stress resilience, sleepiness, type of personality, anxiety and depression in nurses who provide PNC and compare them with those who do not. We hypothesised that nurses who provide PNC are more likely to experience higher occupational stress and have a higher risk of depression and anxiety than those who do not. Associated with some personality traits, nurses' occupational stress during palliative care may impair stress resilience and contribute to higher general feelings of stress and a higher risk for depression and anxiety, creating a dangerous cycle of negative relationships with a higher risk for mental and physical health disorders.

## 4 Methodology

### 4.1 Design

A cross‐sectional study was carried out on nurses employed in healthcare facilities.

### 4.2 Participants

A cross‐sectional survey was conducted on a non‐probabilistic convenience sample of nurses in Croatia. The study took place between April and July 2022. The nurses were recruited through online social media (via nursing groups on Facebook, Instagram, WhatsApp, e‐mails). The online questionnaire in the form of Google Forms was filled out by 185 Croatian nurses. To be eligible for inclusion, criteria were the completion of high medical school and/or nursing studies and employment in healthcare institutions (primary health care, outpatient clinic, hospital, geriatric institution, home care). Exclusion criteria were unemployment, retirement, employment in institutions that are not part of the healthcare system, and an inadequately completed questionnaire. Based on the exclusion criteria, five nurses were excluded from the study. The final sample included 180 nurses. We divided nurses into two study groups: nurses who provide PNC; palliative nurses, PN (*N* = 94) and nurses who do not provide PNC; control group; CG (*N* = 86). Nurses who provide PNC were nurses who provide palliative care every day and several times a week, while CG were nurses who provide palliative care never or rarely.

### 4.3 Ethical Consideration

The study was approved by the Ethics Committee of the University of Zadar, Croatia. At the beginning of the questionnaire, the respondents gave their consent. Before starting to complete the questionnaire, all participants received information about the research objective, procedures to ensure privacy and the option to stop completing the questionnaire at any time. By submitting the consent form (by clicking on the ‘Agree’ button) and completing the questionnaire, the respondents gave their consent to participate in the study. The study was conducted in accordance with the ethical standards of the Declaration of Helsinki.

### 4.4 Questionnaire

The data on sociodemographic data, self‐assessment of personality, self‐esteem, anxiety, depression, sleepiness, resilience and general and occupational nursing stress were measured. The questionnaire consists of a combination of several validated questionnaires, supplemented by a socio‐demographic questionnaire developed specifically for the needs of this study. The questionnaire was designed to be as user‐friendly as possible, with clear instructions and a logical flow. To reduce possible fatigue when completing the questionnaire, we offered participants the opportunity to save their progress and return later if necessary. To avoid duplicate answers, each participant could only complete the questionnaire once.

The *sociodemographic data* questionnaire was developed for the purposes of this study in order to assess sociodemographic, social and health data of the respondents. It included data on gender, age, marital status, parentage, level of education, self‐assessment of economic status, monthly income, number of close friends, frequency of socialising with close friends and self‐assessment of health status, working area, shift work and self‐assessment of own health conditions.

The *Rosenberg Self‐Esteem Scale* (RSES) measures the level of global self‐esteem, more precisely what a person thinks about himself. It contains 10 items, five of which are defined in the negative direction, and these items are scored in reverse. Each response is scored on a four‐point Likert type scale (3 = strongly agree, 2 = agree, 1 = disagree, 0 = strongly disagree). The scores range from 0 to 30 points, and a higher number of points reflects a higher level of self‐esteem (Rosenberg 1965; Schmitt and Allik 2005). The internal consistency of the questionnaire was *α* = 0.73 (from previous studies was *α* = 0.84) (Schmitt and Allik 2005).

The *Ten‐Item Personality Inventory* (TIPI) was used to examine five dimensions of personality: extraversion (extraverted and enthusiastic vs. reserved and quiet), agreeableness (critical and quarrelsome vs. sympathetic and warm), conscientiousness (dependable and self‐disciplined vs. disorganised and careless), emotional stability (anxious and easily upset vs. calm and emotionally stable), openness (open to new experiences and complex vs. conventional and uncreative). It includes 10 items; two items for each personality dimension. Five items are negatively oriented, and the said items were scored in reverse. The total score was obtained by summing the points on a seven‐point Likert scale (1 = do not agree at all; 2 = moderately disagree; 3 = partially disagree; 4 = neither agree nor disagree; 5 = partially agree; 6 = moderately agree; 7 = completely agree) (Gosling et al. 2003). The internal consistency of the questionnaire was *α* = 0.65 (from previous studies was *α* = 0.66) (Tatalović Vokapić 2016).

The *Epworth sleepiness scale* (ESS) was used to assess the level of daytime sleepiness in everyday life situations. It consists of eight items and each response is scored from zero to three (0 = I would never doze; 1 = slight chance doze; 2 = moderate chance of dozing; 3 = high chance of dozing). The total score represents the sum of the points, and the scores range from zero to 24. A score greater than 11 implies an increasing level of excessive daytime sleepiness (Johns 1991; Pecotic et al. 2012). The internal consistency of the questionnaire was *α* = 0.82 (from previous studies was *α* = 0.84) (Pecotic et al. 2012).

The *Generalised Anxiety Disorder* (GAD‐7) was used to determine and assess the degree of symptoms of a generalised anxiety disorder. It consists of seven items and the answers of the respondents refer to the time period of the last 2 weeks. Each answer was scored from zero to three (0 = not at all; 1 = several days; 2 = more than half the days; 3 = nearly every day). The result represents the sum of points on the basis of which the level of anxiety was determined as minimal anxiety (0–4), mild anxiety (5–9), moderate anxiety (10–14) and severe anxiety (15–21) (Spitzer et al. 2006). The internal consistency of the questionnaire was *α* = 0.93 (from previous studies was *α* = 0.87) (Martínez‐Vázquez et al. 2022).

The *Patient Health Questionnaire* (PHQ‐9) measures the presence and severity of depression. It consists of nine items, and respondents' answers refer to the time period of the last 2 weeks. Each response was scored from zero to three (0 = not at all; 1 = several days; 2 = more than half the days; 3 = nearly every day). The result represents the sum of points, and depending on the result, minimal depression (0–4), mild depression (5–9), moderate depression (10–14), moderately severe depression (15–19) and severe depression (20–27) were determined (Kroenke et al. 2001). The internal consistency of the questionnaire was *α* = 0.90 (from previous studies was *α* = 0.83) (Šagud et al. 2023).

The *Brief Resilience Scale* (BRS) examines the relationship between resilience and the ability to recover from stress. It consists of six items, three of which are negatively and three positively phrased. Negatively phrased items were scored in reverse. Each response was scored on a five‐point Likert scale (1 = strongly disagree; 2 = disagree; 3 = neither agree nor disagree; 4 = agree; 5 = strongly agree). The result was obtained by calculating the average score. An average score of < 3.0 indicates low resistance to stress and slower recovery from stressful stimuli, and > 4.3 indicates high resilience and faster recovery from stress (Smith et al. 2008). The internal consistency of the questionnaire was *α* = 0.85 (from previous studies was *α* = 0.82) (Slišković and Burić 2018).

The *Perceived Stress Scale* (PSS‐10) measures the degree to which an individual considers his life unpredictable, under an excessive burden and unable to control. It consisted of 10 items, four of which were positively framed and were scored inversely. Answers were scored on a five‐point Likert scale (0 = never; 1 = almost never; 2 = sometimes; 3 = fairly often; 4 = very often). The result was obtained by adding up the points (ranged from 0 to 40 points), where a higher result indicates that the respondents experience general, everyday stress more difficultly (Cohen et al. 1983; Maroufizadeh et al. 2018). The internal consistency of the questionnaire was *α* = 0.85 (from previous studies was *α* = 0.79) (Nakić et al. 2009).

The *Nursing Stress Scale* (NSS) was used to identify sources of occupational stress and to measure the frequency of exposure of nurses to the aforementioned work stressors. The scale consisted of 34 items divided into seven subscales. The subscales were as follows: death and the process of dying; conflict with the physician; inadequate preparation to deal with the emotional needs of patients and their families; lack of staff support; conflicts with other nurses; workload; uncertainty regarding patient treatment. Respondents assessed on a four‐point scale (1 = never; 2 = occasionally: 3 = often; 4 = very often) how often a certain situation at the workplace was stressful for them. The result represents the sum of points (ranged between 34 to 136 points), where a higher result indicates a higher level of occupational stress of nurses (Gray‐Toft and Anderson 1981). The internal consistency of the questionnaire was *α* = 0.96 (from previous studies was *α* = 0.89) (Alinejad et al. 2023).

### 4.5 Data Analysis

The statistical analysis was conducted using SPSS 25.0 (IBM, Armonk, NY, USA). Statistically significant values were those with *p* < 0.05.

Cronbach's alpha was used to assess the internal consistency of the questionnaires. The Kolmogorov–Smirnov normality test was used to assess data distribution. For numerical variables, the median and interquartile range were used, whereas percentages and absolute numbers were used to describe categorical variables. The chi‐squared test and Mann–Whitney test were used to analyse differences between groups of nurses.

The multivariate linear regression was used to assess associations between main outcome (dependent) variables: occupational nursing stress, depression, anxiety, resilience, perceived general stress, sleepiness, personality and self‐esteem. The main dependent variable in each regression model was palliative nursing care, while nurses' age, gender, education level, marital status, children, income, work area, shift work, health conditions and stress resilience were additional independent variables of interest.

We used G*Power v3.1.9.4 software (Universität Kiel, Kiel, Germany) to perform a priori power analysis (Faul et al. 2007). Based on 22 predictors (10 sociodemographic and 12 psychological characteristic) in multiple linear regressions, with medium effect size *f* ^2^ = 0.15, power 1 − *β* = 0.95 and significance level *α* = 0.05, it yielded a required sample of 75 nurses. A posteriori analysis on a sample of 180 nurses yielded satisfactory power (1 − *β* = 0.99).

## 5 Results

### 5.1 Sociodemographic Characteristics of Study Groups

Both groups of nurses had similar sociodemographic characteristics. Nurses in the CG were a borderline insignificantly older (Mdn = 40.0, IQR = 20.0) than PN (Mdn = 36.5, IQR = 15.0); *p* = 0.070. Female gender, married nurses who have children, bachelor's degree, working in a hospital, shift work and good self‐assessment of own health were dominant. No differences were found between groups in sociodemographic characteristics (Table 1).

**TABLE 1:** Sociodemographic characteristics of study group (*N* = 180).

	Palliative care nurses (*n* = 94)	Control nurses' group (*n* = 86)	*p*
Gender; *N* (%)			
Female	92 (98.9)	82 (95.3)	0.147^a^
Male	1 (1.1)	4 (4.7)	
Age; Mdn (IQR)	36.5 (15.0)	40.0 (20.0)	0.070^b^
Age groups, *N* (%)			
≤ 25	19 (20.2)	16 (18.6)	0.144^a^
26–35	23 (24.5)	15 (17.4)	
36–45	34 (36.2)	24 (27.9)	
46–55	14 (14.9)	23 (26.7)	
≥ 56	4 (4.3)	8 (9.3)	
Marital status, *N* (%)			
Married, live together	71 (75.5)	58 (67.4)	0.229^a^
Alone, divorce, widow	23 (24.5)	28 (32.6)	
Children, *N* (%)			
Yes	60 (63.8)	60 (69.8)	0.399^a^
No	34 (36.2)	26 (30.2)	
Education, *N* (%)			
High school	31 (33.0)	21 (24.4)	0.239^a^
Bachelor degree	41 (43.6)	36 (41.9)	
Master degree	22 (23.4)	29 (33.7)	
Monthly income, *N* (%)			
> 1000€	79 (84.0%)	71 (82.6)	0.790^a^
< 1000€	15 (16.0%)	15 (17.4)	
Working area, *N* (%)			
Primary, outpatient clinic	18 (19.1)	14 (16.3)	0.212^a^
Hospital work	64 (68.1)	67 (77.9)	
Geriatric; home care	12 (12.8)	5 (5.8)	
Shift working, *N* (%)			
One shift (morning)	41 (43.6)	40 (46.5)	0.697^a^
Three shifts	53 (56.4)	46 (53.5)	
Self‐perception own health, *N* (%)			
Bad conditions	3 (3.2)	5 (5.8)	0.394^a^
Good conditions	91 (96.8)	81 (94.2)	

^a^ Chi squared test.

^b^ Mann–Whitney test.

### 5.2 Psychological Characteristics of Study Groups

No differences were found between groups in self‐esteem, anxiety, depression, sleepiness, resilience, general stress and personality such as extraversion, agreeableness, conscientiousness, emotional stability and openness. PN displayed on average the higher of nurses' occupational stress (Mdn = 49.0; IQR = 29.0) when compared with CG (Mdn = 34.0; IQR = 23.0), *p* < 0.001. Exposure to death and dying (*p* < 0.001), conflict with physicians (*p* < 0.001), inadequate preparation (*p* < 0.001), conflict with nurses (*p* = 0.042), workload (*p* = 0.011) and uncertainty about treatment (*p* < 0.001) were higher in PN (Table 2).

**TABLE 2:** Differences between nurses' personality, anxiety, depression and occupational stress perception between study groups (*N* = 180).

	Palliative care nurses (*n* = 94)	Control nurses' group (*n* = 86)	*p*
*Self‐esteem*			
Mdn (IQR)	20.0 (6.0)	21.0 (6.0)	0.230^a^
Mean Rank	86.1	95.4	
*Nurses personality*			
Extraversion			
Mdn (IQR)	5.5 (2.5)	5.3 (2.0)	0.859^a^
Mean Rank	89.8	91.2	
Agreeableness			
Mdn (IQR)	5.5 (1.6)	5.5 (2.0)	0.754^a^
Mean Rank	89.3	91.8	
Conscientiousness			
Mdn (IQR)	6.5 (2.0)	6.5 (1.5)	0.239^a^
Mean Rank	86.3	95.1	
Emotional stability			
Mdn (IQR)	4.5 (1.5)	4.8 (2.0)	0.197^a^
Mean Rank	85.7	95.7	
Openness			
Mdn (IQR)	5.5 (1.6)	5.0 (1.5)	0.529^a^
Mean Rank	92.8	88.0	
*Sleepiness*			
Mdn (IQR)	7.0 (6.0)	8.0 (7.0)	0.185^a^
Mean Rank	85.6	95.9	
*Anxiety*			
Mdn (IQR)	6.0 (6.0)	7.0 (6.0)	0.994^a^
Mean Rank	90.5	90.5	
*Depression*			
Mdn (IQR)	5.0 (9.0)	5.0 (7.0)	0.634^a^
Mean Rank	92.3	88.6	
*Perceived general stress*			
Mdn (IQR)	18.5 (10.0)	17.5 (8.0)	0.320^a^
Mean Rank	94.2	86.5	
*Stress resilience*			
Mdn (IQR)	3.5 (1.0)	3.5 (1.0)	0.667^a^
Mean Rank	92.1	88.8	
*Occupational nursing stress*			
Exposure to death and dying			
Mdn (IQR)	14.0 (7.0)	8.0 (7.0)	< 0.001^a^
Mean Rank	113.9	64.9	
Conflict with physicians			
Mdn (IQR)	5.0 (5.0)	3.0 (3.0)	< 0.001^a^
Mean Rank	105.9	73.7	
Inadequate preparation			
Mdn (IQR)	4.0 (3.0)	3.0 (3.0)	< 0.001^a^
Mean Rank	106.5	73.0	
Lack of staff support			
Mdn (IQR)	3.0 (4.0)	3.0 (4.0)	0.187^a^
Mean Rank	95.3	85.3	
Conflict with other nurses			
Mdn (IQR)	5.0 (5.0)	4.0 (3.0)	0.042^a^
Mean Rank	98.0	82.3	
Exposure to work load			
Mdn (IQR)	11.0 (6.0)	9.5 (6.0)	0.011^a^
Mean Rank	99.9	80.2	
Uncertainty concerning treatment			
Mdn (IQR)	7.0 (5.0)	5.0 (4.0)	< 0.001^a^
Mean Rank	105.9	73.7	
Nursing stress (overall)			
Mdn (IQR)	49.0 (29.0)	34.0 (23.0)	< 0.001^a^
Mean Rank	108.9	70.4	

Abbreviations: IQR, Interquartile Range; Mdn, median.

^a^ Mann–Whitney test.

### 5.3 Associations Between Providing Palliative Nursing Care and Psychological Measures

The linear regression confirmed the positive association between providing PNC and nursing stress (*β* = 0.36; *p* < 0.001), while the negative association was found with daily sleepiness (*β* = −0.19; *p* = 0.021). Marital status was associated with conscientiousness (*β* = 0.18; *p* = 0.028), while education was negatively associated with extraversion (*β* = −0.15; *p* = 0.044). Monthly income contributes to perceived general stress (*β* = 0.15; *p* = 0.006). Perceived general stress was positively associated with depression (*β* = 0.21; *p* = 0.007), anxiety (*β* = 0.30; *p* < 0.001) and negatively with resilience (*β* = −0.22; *p* = 0.011) and emotional stability (*β* = −0.20; *p* = 0.036). Depression was associated with occupational stress (*β* = 0.27; *p* = 0.009), anxiety (*β* = 0.35; *p* < 0.001) and sleepiness (*β* = 0.29; *p* = 0.014). Additionally, resilience was positively associated with self‐esteem (*β* = 0.19; *p* = 0.038) and extraversion (*β* = 0.18; *p* = 0.046) and negatively with anxiety (*β* = −0.27; *p* = 0.005) and agreeableness (*β* = −0.19; *p* = 0.011). Nurses' occupational stress was associated with depression (*β* = 0.16; *p* = 0.009) and anxiety (*β* = 0.12; *p* = 0.043) (Table 3).

**TABLE 3:** Associations between providing palliative nursing care, sociodemographic characteristics and psychological indices using linear regression models.

	Nursing occupational stress		Depression		Anxiety		Resilience		Perceived general stress		Sleepiness		Openness		Emotional stability		Conscientiousness		Agreeableness		Extraversion		Self‐esteem	
	*β*	*p*	*β*	*p*	*β*	*p*	*β*	*p*	*β*	*p*	*β*	*p*	*β*	*p*	*β*	*p*	*β*	*p*	*β*	*p*	*β*	*p*	*β*	*p*
Palliative nursing care (no providing PNC was referent group)																								
Providing PNC	0.36	< 0.001	−0.03	0.539	−0.08	0.107	0.06	0.326	0.06	0.281	−0.19	0.021	0.043	0.580	−0.05	0.481	−0.06	0.410	0.04	0.586	−0.04	0.624	−0.07	0.353
Age	0.01	0.895	−0.03	0.632	−0.05	0.446	−0.09	0.196	−0.02	0.817	0.10	0.308	−0.124	0.163	0.13	0.107	−0.09	0.272	−0.08	0.359	0.10	0.249	0.15	0.086
Gender (male referent group)																								
Female	0.01	0.921	−0.03	0.590	0.03	0.576	0.03	0.606	0.01	0.806	0.09	0.215	−0.017	0.811	0.04	0.539	0.02	0.774	0.00	0.960	0.08	0.217	−0.04	0.516
Marital status (married referent group)																								
No marriage	0.10	0.196	−0.03	0.610	0.09	0.128	0.14	0.052	0.03	0.633	−0.01	0.909	−0.106	0.220	−0.09	0.274	0.18	0.028	0.02	0.857	−0.09	0.249	0.09	0.271
Children (without children referent group)																								
Children (yes)	−0.02	0.799	−0.02	0.703	−0.05	0.368	−0.11	0.146	−0.03	0.667	−0.07	0.490	−0.028	0.755	−0.07	0.396	−0.09	0.303	0.08	0.374	0.08	0.366	−0.07	0.400
University (no university was referent group)																								
University (yes)	−0.03	0.714	−0.02	0.758	0.06	0.245	0.07	0.273	−0.07	0.249	0.03	0.692	0.058	0.466	−0.01	0.941	0.12	0.109	0.06	0.476	−0.15	0.044	0.09	0.226
Income (> 1000€ was referent group)																								
< 1000€	0.02	0.751	−0.01	0.916	0.03	0.552	0.10	0.078	0.15	0.006	−0.10	0.188	−0.075	0.301	0.04	0.547	−0.04	0.525	−0.03	0.687	0.04	0.556	0.02	0.790
Working area (primary, ambulance is referent group)																								
Hospital work	0.13	0.105	0.07	0.255	0.00	0.935	0.02	0.762	−0.03	0.626	−0.12	0.218	−0.013	0.879	−0.05	0.498	0.13	0.121	−0.05	0.601	−0.07	0.413	−0.12	0.147
Geriatric; home care	0.09	0.240	0.01	0.867	−0.05	0.413	0.02	0.783	0.05	0.438	0.03	0.750	−0.027	0.750	−0.09	0.243	0.04	0.637	0.20	0.014	0.01	0.926	−0.02	0.810
Shift working (no was referent group)																								
Shift working (yes)	−0.01	0.864	−0.02	0.762	−0.08	0.153	−0.14	0.055	−0.02	0.740	0.02	0.845	0.057	0.513	0.07	0.380	−0.11	0.191	0.01	0.895	−0.01	0.933	0.10	0.224
Health conditions (good health was referent group)																								
Bad health	−0.02	0.795	−0.02	0.687	0.10	0.039	0.00	0.965	0.00	0.978	0.00	0.967	0.080	0.270	−0.01	0.912	0.09	0.194	−0.09	0.238	−0.09	0.175	0.02	0.822
Self‐esteem	0.00	0.961	−0.02	0.787	−0.02	0.699	0.14	0.038	−0.08	0.184	−0.04	0.676	0.112	0.170	0.04	0.597	0.02	0.776	0.08	0.310	0.11	0.161	—	—
Extraversion	0.08	0.348	−0.06	0.355	0.07	0.228	0.14	0.046	−0.01	0.821	−0.04	0.658	0.352	< 0.001	0.13	0.088	0.24	0.002	−0.09	0.291	—	—	0.12	0.161
Agreeableness	−0.10	0.201	0.00	0.995	0.03	0.557	−0.13	0.043	0.02	0.697	−0.02	0.823	0.011	0.887	0.32	< 0.001	0.20	0.008	—	—	−0.08	0.291	0.08	0.310
Conscientiousness	0.00	0.998	−0.06	0.295	−0.05	0.343	−0.07	0.353	0.02	0.808	0.06	0.484	0.241	0.004	0.14	0.073	—	—	0.22	0.008	0.24	0.002	0.02	0.776
Emotional stability	0.05	0.537	0.00	0.994	−0.05	0.399	0.13	0.071	−0.14	0.036	−0.02	0.839	−0.090	0.309	—	—	0.15	0.073	0.39	< 0.001	0.14	0.088	0.05	0.597
Openness	0.07	0.350	−0.02	0.764	−0.01	0.918	0.00	0.985	0.03	0.642	0.04	0.658	—	—	−0.07	0.309	0.21	0.004	0.01	0.887	0.31	< 0.001	0.11	0.170
Sleepiness	0.09	0.192	0.13	0.014	0.02	0.692	0.00	0.973	−0.01	0.850	—	—	0.033	0.658	−0.01	0.839	0.05	0.484	−0.02	0.823	−0.03	0.658	−0.03	0.676
Perceived general Stress	−0.06	0.576	0.21	0.007	0.30	< 0.001	−0.22	0.011	—	—	−0.02	0.850	0.050	0.642	−0.20	0.036	0.02	0.808	0.04	0.697	−0.02	0.821	−0.14	0.184
Resilience	−0.08	0.363	−0.06	0.406	−0.18	0.005	—	—	−0.18	0.011	0.00	0.973	−0.002	0.985	0.16	0.071	−0.08	0.353	−0.19	0.043	0.18	0.046	0.19	0.038
Anxiety	0.22	0.043	0.40	< 0.001	—	—	−0.27	0.005	0.35	< 0.001	0.05	0.692	−0.012	0.918	−0.09	0.399	−0.11	0.343	0.07	0.557	0.13	0.228	−0.04	0.699
Depression	0.27	0.009	—	—	0.35	< 0.001	−0.08	0.406	0.22	0.007	0.29	0.014	−0.033	0.764	0.00	0.994	−0.11	0.295	0.00	0.995	−0.10	0.355	−0.03	0.787
Nursing occupational stress	—	—	0.16	0.009	0.12	0.043	−0.06	0.363	−0.04	0.576	0.12	0.192	0.080	0.350	0.05	0.537	0.00	0.998	−0.11	0.201	0.07	0.348	0.00	0.961

Abbreviations: *β*, beta coefficient; *p*, *p* value; PNC, palliative nursing care.

### 5.4 Associations Between Providing Palliative Nursing Care and Domain Nursing Stress Scale

The nurses who provide PNC had a higher perception of stressful situations as a result of suffering, dying and death (*β* = 0.28; *p* < 0.001), a higher feeling of inadequate preparation to deal with the emotional needs of the patients (*β* = 0.14; *p* = 0.022) and a lack staff support (*β* = −0.14; *p* = 0.028). Death and dying were associated with conscientiousness (*β* = 0.15; *p* = 0.026). Inadequate preparation was negatively associated with older age (*β* = −0.16; *p* = 0.016) and resilience (*β* = −0.18; *p* = 0.019) and positively associated with geriatric care and home care (*β* = 0.13; *p* = 0.021). Conflict with the physician was associated with nurses' agreeableness (*β* = 0.12; *p* = 0.031), openness (*β* = 0.10; *p* = 0.050), resilience (*β* = 0.18; *p* = 0.006) and anxiety (*β* = 0.21; *p* = 0.007). Lack of support was positively associated with anxiety (*β* = 0.20; *p* = 0.034) and had a negative contribution to emotional stability (*β* = −0.24; *p* = 0.001). Conflicts with nurses were negatively associated with shift work (*β* = −0.12; *p* = 0.049), agreeableness (*β* = −0.20; *p* = 0.001), anxiety (*β* = −0.25; *p* = 0.002) and resilience (*β* = −0.22; *p* = 0.004), while no marriage positively contributed (*β* = 0.16; *p* = 0.014). Workload was associated with work at hospitals (*β* = 0.15; *p* = 0.023) and openness (*β* = 0.12; *p* = 0.044), while uncertainty regarding patient treatment was associated with agreeableness (*β* = −0.12; *p* = 0.031) and emotional stability (*β* = 0.12; *p* = 0.041).

The linear regression confirmed the associations between domains. Death and dying were contributed to workload (*β* = 0.23; *p* = 0.006) and uncertainty regarding patient treatment (*β* = 0.23; *p* = 0.014). Conflict with the physician contributes to conflicts with other nurses (*β* = 0.49; *p* < 0.001) and uncertainty regarding patient treatment (*β* = 0.35; *p* < 0.001), and vice versa. There were associations between lack of support and uncertainty concerning the treatment of patients (*β* = 0.41; *p* < 0.001). Workload, inadequate preparation and uncertainty concerning treatment contribute to the majority domain (Table 4).

**TABLE 4:** Associations between providing palliative nursing care and the domain nursing stress scale using linear regression models.

	Death and dying		Conflict with physicians		Inadequate preparation		Lack of staff support		Conflict with other nurses		Work load		Uncertainty concerning treatment	
	*β*	*p*	*β*	*p*	*β*	*p*	*β*	*p*	*β*	*p*	*β*	*p*	*β*	*p*
Palliative nursing care (no providing PNC was referent group)														
Providing PNC	0.28	< 0.001	0.04	0.486	0.14	0.022	−0.14	0.028	−0.02	0.683	−0.08	0.175	0.08	0.168
Age	0.06	0.420	0.07	0.251	−0.16	0.016	0.10	0.169	0.01	0.912	0.05	0.480	−0.09	0.112
Gender (male referent group)														
Female	0.06	0.270	0.03	0.495	0.03	0.596	−0.05	0.403	0.02	0.695	−0.03	0.497	−0.04	0.444
Marital status (married referent group)														
No marriage	0.05	0.472	−0.01	0.881	−0.06	0.326	−0.03	0.697	0.16	0.014	−0.09	0.174	0.04	0.446
Children (without children referent group)														
Children (yes)	−0.04	0.558	0.01	0.914	0.02	0.813	0.07	0.351	−0.09	0.157	0.06	0.340	−0.02	0.677
University (no university was referent group)														
University (yes)	−0.06	0.340	0.03	0.538	0.05	0.434	−0.09	0.134	0.04	0.509	0.01	0.844	−0.01	0.777
Income (> 1000€ was referent group)														
< 1000€	−0.02	0.721	0.01	0.817	−0.02	0.732	0.13	0.021	−0.06	0.289	−0.02	0.691	0.00	0.923
Working area (primary. ambulance is referent group)														
Hospital work	−0.08	0.252	0.01	0.879	−0.02	0.801	−0.02	0.762	0.04	0.498	0.15	0.023	−0.02	0.756
Geriatric and home care	−0.06	0.374	0.02	0.717	0.13	0.034	−0.03	0.636	0.03	0.624	−0.07	0.233	0.04	0.422
Shift working (no was referent group)														
Shift working (yes)	0.01	0.920	0.08	0.173	0.00	0.978	−0.01	0.934	−0.12	0.049	0.06	0.329	−0.04	0.541
Health conditions (good was referent group)														
Bad conditions	0.00	0.956	0.01	0.757	0.02	0.766	−0.08	0.159	−0.05	0.364	0.01	0.844	0.04	0.397
Self‐esteem	0.01	0.909	−0.10	0.055	−0.04	0.554	0.12	0.077	0.07	0.232	0.04	0.555	−0.04	0.457
Extraversion	0.04	0.505	−0.07	0.237	0.05	0.423	−0.05	0.433	0.11	0.067	−0.07	0.289	0.04	0.447
Agreeableness	0.01	0.821	0.12	0.031	−0.10	0.101	0.12	0.056	−0.20	0.001	0.11	0.065	−0.12	0.031
Conscientiousness	0.15	0.026	−0.01	0.869	−0.11	0.085	0.10	0.137	−0.07	0.284	0.07	0.303	−0.08	0.153
Emotional stability	−0.12	0.103	−0.02	0.785	0.11	0.110	−0.24	0.001	0.09	0.175	0.00	0.997	0.12	0.041
Openness	0.02	0.757	0.10	0.050	−0.04	0.488	−0.02	0.731	−0.09	0.134	0.12	0.044	−0.06	0.260
Sleepiness	−0.02	0.753	−0.01	0.767	0.07	0.199	−0.08	0.154	0.04	0.501	0.01	0.832	0.04	0.378
Perceived general Stress	−0.06	0.491	−0.05	0.462	−0.07	0.385	−0.01	0.900	0.10	0.213	0.12	0.147	−0.07	0.360
Resilience	−0.08	0.287	0.18	0.006	−0.18	0.019	0.05	0.496	−0.22	0.002	0.11	0.145	0.00	0.947
Anxiety	−0.03	0.794	0.21	0.007	−0.08	0.370	0.20	0.034	−0.25	0.004	0.07	0.457	0.00	0.976
Depression	0.05	0.558	0.07	0.332	0.14	0.085	−0.08	0.367	0.09	0.269	−0.10	0.216	0.03	0.702
Death and dying	—	—	0.09	0.189	0.10	0.196	0.06	0.469	−0.05	0.508	0.21	0.006	0.17	0.014
Conflict with physicians	0.13	0.189	—	—	0.27	0.003	−0.07	0.459	0.49	< 0.001	−0.15	0.096	0.35	< 0.001
Inadequate preparation	0.11	0.196	0.21	0.003	—	—	0.30	< 0.001	−0.05	0.520	0.35	< 0.001	−0.14	0.047
Lack of staff support	0.06	0.469	−0.05	0.459	0.27	< 0.001	—	—	0.27	< 0.001	−0.14	0.071	0.29	< 0.001
Conflict with other nurses	−0.06	0.508	0.41	< 0.001	−0.05	0.520	0.32	< 0.001	—	—	0.27	0.001	−0.04	0.555
Work load	0.23	0.006	−0.12	0.096	0.36	< 0.001	−0.16	0.071	0.25	0.001	—	—	0.36	< 0.001
Uncertainty concerning treatment	0.23	0.014	0.34	< 0.001	−0.18	0.047	0.41	< 0.001	−0.05	0.555	0.44	< 0.001	—	—

Abbreviations: *β*, beta coefficient; *p*, *p* value; PNC, palliative nursing care.

## 6 Discussion

The aim of this study was to investigate the level of occupational stress in nurses providing PNC. Our results confirm that nurses who provide PNC have higher levels of nurses' occupational stress than nurses who do not provide PNC. Palliative care has been associated with nursing stress, death and dying, inadequate preparation to meet the emotional needs of patients and their families, lack of support and sleepiness. Lack of support was related to uncertainty about care, which has been associated with death and dying, conflict with the physician and other nurses, inadequate preparation and workload. Stress in nursing has been associated with anxiety and depression, and vice versa. The relationship between occupational stress, anxiety and depression, as well as their association with general stress, sleepiness and resilience, has been noted in other studies (Cross 2019). These can be explained by the patients' illness and long suffering. Nurses who are confronted with the dying and death of patients may experience anxiety, fear and depression, which negatively affect quality of life, and are at risk of burnout syndrome (Cross 2019; May et al. 2022). In addition, nursing stress is often associated with the care provided to the patient, inability to meet patients' needs and inadequate communication with patients and family members, and lower patient safety (Babapour et al. 2022; Kwame and Petrucka 2021; Tamata and Mohammadnezhad 2023). A shortage of nursing staff, a lack of time, a high workload and fatigue are some of the complex barriers that result in fewer interactions between nurses, patients and caregivers and higher stress perception (Kwame and Petrucka 2021).

The association of death and dying with nurses' stress may be explained by the special circumstances of caring for critically ill patients, the proximity to the end of life, and the constant empathy of nurses (Babapour et al. 2022; Wilkinson et al. 2017). This can be caused by the inability to control the patient's pain and other unpleasant symptoms, which can create a feeling of helplessness and the futility of interventions. Additionally, avoiding conversations about death may also contribute to nurses' anxiety and increase patients' fear and sense of loneliness (Ramvi et al. 2021). Furthermore, our results show that perceived general stress and anxiety were associated with lower resilience. These associations can lead to emotional exhaustion, depersonalization, and a sense of lower personal accomplishment (Zanatta et al. 2020). On the other hand, resilience in the context of positive effects on stress can reduce stress perception and increase well‐being and mental health (Zanatta et al. 2020). Our results also confirm that higher resilience was associated with higher self‐esteem and extraversion. These could be important coping strategies to reduce stress levels and burnout syndrome in nurses. In addition, resilience was positively related to self‐esteem and extraversion but negatively related to agreeableness. Agreeableness as a determinant of prosocial behaviour includes compassion, empathy, compliance, altruism and trust. It is possible that consistently high levels of compassion and empathy contribute to emotional exhaustion, which negatively affects resilience. While empathy is important for effective care, it can make nurses vulnerable to stress‐related conditions such as compassion fatigue and emotional exhaustion (Wilkinson et al. 2017). Also, agreeableness may be associated with cognitive constructs such as self‐regulation and effortful control and induce anxiety and depression (Lyon et al. 2021; Ode and Robinson 2007). In our study, caregiving stress was associated with anxiety, which negatively affected resilience.

Our results indicate that a significant source of nurses' stress can be inadequate preparation to meet patients' emotional needs and unsatisfactory responses to the questions asked. This could be attributed to the lack of specific communication skills and training to provide emotional and psychological support to the patient. For this reason, nurses may feel a sense of insecurity, coupled with the fear of expressing themselves incorrectly or giving false information. Therefore, acquiring specific communication skills would likely reduce nurses' stress (Aryankhesal et al. 2019; Swain and Gale 2014). In addition, there is uncertainty about treatment and care, likely due to incomplete information, inadequate organisation, interventions that contribute to patient suffering and uncertainty about the use of specialised equipment. These stressors are often the result of inadequate communication among team members, unclear responsibilities and lack of skills and training (Ellen et al. 2021). Studies highlighting the importance of educating nurses on issues related to death and dying point to the emphasis on workshops, classes, active discussions and the presentation of personal attitudes to relieve occupational stress (Guo and Zheng 2019).

Relationships and conflicts with physicians and other nurses are also a major contributor to stress in nursing. Studies show that other professionals have different perceptions about the role of nurses' in palliative care and conflicting opinions about best practice in end‐of‐life care (Tong et al. 2022). These reasons and the patients' interests may be a cause for discussion among team members. The increased workload in the hospital is not surprising, considering that palliative care is still developing, and therefore there are certain implementation difficulties (Fadhil et al. 2017; Vočanec et al. 2022). For example, in the absence of better options, patients requiring palliative care are often placed in acute care hospitals (Paes et al. 2018). Although the number of palliative care beds and palliative care units has increased, there is still a shortage of nurses in general and a shortage of nurses with specialised palliative care training (Vočanec et al. 2022). Regular curative treatments for acute patients, staff shortages, a heavy workload of administrative tasks for nurses, and unpredictable shift changes when moving to a new work environment are just some of the stressful factors. Nursing staff are overworked to provide the best possible care, while patients don't receive comprehensive palliative care, as acute hospitals aren't primarily focused on incurable illnesses (Tamata and Mohammadnezhad 2023; Vočanec et al. 2022). In addition, these factors leave nurses with insufficient time to take a holistic approach, which is of vital importance for patients because it is focused on the whole person, not just the disease. Also, insufficient nursing emotional support to patients and their familiescan lead to higher levels of perceived occupational stress in nurses (Paes et al. 2018). Furthermore, it is possible that a constant workload interferes with nurses' daily personal lives and leads to increased sensitivity to everyday stressors. This has additional negative effects on family and individual quality of life. This is confirmed by other studies that emphasise that a major cause of general stress is workplace stress, which can have negative effects on the individual's physical and mental health, behaviour, and also private life (Abdoh et al. 2021).

Greater focus on patient needs, and increased stress may contribute to constant tension, insomnia and reduced sleepiness, which confirms an association between the provision of PCN and reduced daily sleepiness in our study. Also, permanent tension caused by the patient's perception of stress, anxiety and dying does not allow sleep despite pronounced fatigue (Kalmbach et al. 2018). It can be challenging for the physician, nurse, patient, family, or healthcare proxy to decide whether to use procedures to extend the patient's life or provide comfort during the final stages of their treatment (Akdeniz et al. 2021). Many of the contextual ethical issues that palliative care practitioners face, such as resolving inter‐professional conflict, institutional policies and resource allocation, are not covered in training on palliative care resulting in higher stressful perception (Schofield et al. 2021). In addition, the stress response is extremely complex, which is reflected in the various reactions. Circadian rhythms disrupted by shift work can also significantly affect wakefulness. Response to stress often manifests as a sleep disturbance, known as sleep reactivity (Kalmbach et al. 2018). Consequently, people with a highly reactive sleep system are more likely to develop insomnia after a stressor, which increases the risk of psychopathology and can hinder recovery from traumatic stress (Reffi et al. 2023). Nurses with higher sleep reactivity may have been physiologically overexcited throughout the day, which is confirmed by some studies (Kalmbach et al. 2018; Shi et al. 2021).

### 6.1 Strengths and Limitation

This work contributes to the existing literature on this vulnerable and challenging area to clarify some issues related to palliative nursing care. First, the palliative care nurses are vulnerable to the negative consequences of prolonged psychological stress. Indeed, the study confirms the association between occupational stress, anxiety, depression, perceived general stress, resilience and personality, which may have negative repercussions on the nurses' physical and psychological well‐being, also reflecting on the quality of PNC provided as well as the quality of the health care.

Despite these advantages, this study has some limitations. First, it is a cross‐sectional study, and it is not possible to establish causality of associations between PNC provision and caregiving stress or other psychological and personal factors. Nevertheless, this study may provide new hypotheses for future research. Second, although statistically significant, the relatively small sample size may have influenced some associations between variables. However, we were unable to recruit more nurses. Thirdly, some of the nurses did not work all three shifts and worked in different working areas, which may have affected sleep assessment, understanding of working conditions in palliative care and stressors faced by nurses. Nevertheless, this study is a valuable scientific contribution because it demonstrates the importance of developing strategies to improve nurses' mental and psychological health. Finally, palliative care includes pain management, psychological support, end‐of‐life care and other elements, so it could be said that all nurses in hospitals provide palliative care on a daily basis. Despite that, this can have a positive impact on the provision of palliative care in facilities that don't specialise in palliative care.

### 6.2 Recommendations for Further Research and Implications for Practice

As palliative care is essential at a time when chronic and other terminal illnesses are on the rise, future studies should identify which elements of the work environment promote occupational stress. It would be useful to investigate how to improve working conditions, how occupational stress affects nurses' professional development and job satisfaction, and to what extent it negatively impacts the overall quality of care.

Public government should focus on interventions that can have an impact on reducing occupational stress for all caregivers, especially those in palliative care. These include providing psychological support to nurses, stress management, respecting their autonomy, ensuring better working conditions, attending relevant courses, spiritual practices and ensuring lifelong learning (Clayton and Marczak 2023; Harder et al. 2020; Lowe et al. 2016; Sapeta et al. 2022; Zheng et al. 2018). Empowering nurses' autonomy can not only help to influence nurses' attitudes towards caring for dying patients (Miyashita et al. 2007). Nurses' autonomy is a crucial factor in palliative care as it is related to nurses' perception of the importance of the nurses' profession. Autonomy is linked with expertise, clinical judgement, the ability to influence work processes, participate in decision‐making, and to nurses' acting publicly (Peacock and Hernandez 2020; Pursio et al. 2021). In addition, nurses' autonomy may reduce the perception of stress by giving nurses a greater sense of satisfaction and competence in solving problems within the scope of their responsibilities (Miyashita et al. 2007; Peacock and Hernandez 2020; Pursio et al. 2021). A healthcare system that promotes lifelong learning through specific education palliative care programmes is crucial as it can prepare caregivers for the challenges associated with palliative and end‐of‐life care. Effective integration and use of relevant data from many medical specialties and databases is essential for palliative care (Currow and Abernethy 2005). Coping strategies in palliative care include personal and professional growth and development, debriefing, speaking up and being heard, better understanding of ethical principles, promoting communication and collaboration among members of the multidisciplinary team, perfecting specific communication and practical skills, and separating personal and environmental factors (Clayton and Marczak 2023; Harder et al. 2020; Lowe et al. 2016; Sapeta et al. 2022; Zheng et al. 2018). Although respite care is always talked about in the context of family members, the concept of respite care could be a great help in empowering caregivers and reducing stress (Rao et al. 2021).

## 7 Conclusion

Findings from our study reveal that an association between the provision of PNC and nursing occupational stress in caregiving suggests a potential link with negative health outcomes for nurses. These findings highlight a particular challenge to the functioning of the healthcare system. Therefore, public health programs are essential to improve nurses' mental health, strengthen personal skills, and reduce nursing stress. Nurses would benefit from psychological support to develop coping skills, improve working conditions, promote their autonomy and receive continuing education. These preventive strategies will slow the deterioration of mental and physical health of nurses caring for patients who require palliative care. This will contribute to higher patients' satisfaction, positive effectiveness and quality of palliative care at all levels of the healthcare system.

## Author Contributions

All authors had access to the data and a role in the writing of the manuscript, selection of the article type, key words and running head. Author's contribution: M.L. and G.P. conceived and designed this study; M.L., G.P., I.G., S.Š., B.D., M.M.S., T.Š., S.Č., D.N. and S.K. acquired the data. M.L. analysed and interpreted the data. M.L. and G.P. wrote the first draft of the paper. All authors participated in drafting the final version of the paper and revised it critically for important intellectual content. All authors approved the final version of the submitted manuscript.

## Ethics Statement

The study was approved by the Human Research Ethics Committee of the University of Zadar (114‐06/22‐01/20; 15‐22‐01). The research was in compliance with the ethical standards of the Declaration of Helsinki.

## Conflicts of Interest

The authors declare no conflicts of interest.

## Data Availability

Data sharing upon reasonable request.
